# Supplementary material for: RA-induced prominence-specific response resulted in distinctive regulation of Wnt and osteogenesis
Source: Life Sci Alliance. 2023 Aug 4;6(10):e202302013. doi: 10.26508/lsa.202302013 (PMC10403638; doi:10.26508/lsa.202302013)
Supplement: Supplementary file 4 [file LSA-2023-02013_TableS4.doc]

**Supplemental Table S4** Gene Ontology Biological Process terms enriched in genes differentially expressed by Prominence.

| **GO Biological Process Term** | **Count** | **GeneRatio** | **Fold.Enrich** | **p.adjust** |
| --- | --- | --- | --- | --- |
| GO:0045944~positive regulation of transcription from RNA polymerase II promoter | 51 | 10.03937 | 1.816289 | 0.008293 |
| GO:0007275~multicellular organism development | 49 | 9.645669 | 1.937896 | 0.004762 |
| GO:0007155~cell adhesion | 48 | 9.448819 | 3.452973 | 3.95E-10 |
| GO:0030154~cell differentiation | 43 | 8.464567 | 1.822074 | 0.025612 |
| GO:0030198~extracellular matrix organization | 35 | 6.889764 | 8.237561 | 9.21E-18 |
| GO:0016310~phosphorylation | 31 | 6.102362 | 2.141128 | 0.018526 |
| GO:0006468~protein phosphorylation | 31 | 6.102362 | 2.049599 | 0.031858 |
| GO:0035556~intracellular signal transduction | 29 | 5.708661 | 2.790833 | 0.001052 |
| GO:0001525~angiogenesis | 26 | 5.11811 | 3.964637 | 1.11E-05 |
| GO:0016477~cell migration | 22 | 4.330709 | 3.123714 | 0.003702 |
| GO:0007507~heart development | 21 | 4.133858 | 3.051768 | 0.006127 |
| GO:0007156~homophilic cell adhesion via plasma membrane adhesion molecules | 20 | 3.937008 | 5.035585 | 1.31E-05 |
| GO:0006366~transcription from RNA polymerase II promoter | 19 | 3.740157 | 3.471792 | 0.003785 |
| GO:0030335~positive regulation of cell migration | 19 | 3.740157 | 3.116722 | 0.007518 |
| GO:0001666~response to hypoxia | 17 | 3.346457 | 3.573799 | 0.006428 |
| GO:0007411~axon guidance | 16 | 3.149606 | 3.68562 | 0.007518 |
| GO:0098609~cell-cell adhesion | 16 | 3.149606 | 3.363575 | 0.013844 |
| GO:0016055~Wnt signaling pathway | 16 | 3.149606 | 2.923614 | 0.039056 |
| GO:0006816~calcium ion transport | 14 | 2.755906 | 4.096517 | 0.007518 |
| GO:0042060~wound healing | 12 | 2.362205 | 4.767637 | 0.007518 |
| GO:0030199~collagen fibril organization | 11 | 2.165354 | 8.8216 | 0.000213 |
| GO:0045785~positive regulation of cell adhesion | 10 | 1.968504 | 6.014727 | 0.007518 |
| GO:0071277~cellular response to calcium ion | 10 | 1.968504 | 4.811782 | 0.026382 |
| GO:0070588~calcium ion transmembrane transport | 10 | 1.968504 | 4.55853 | 0.0342 |
| GO:0031532~actin cytoskeleton reorganization | 9 | 1.771654 | 5.99622 | 0.016563 |
| GO:0002062~chondrocyte differentiation | 9 | 1.771654 | 5.817229 | 0.018526 |
| GO:0001568~blood vessel development | 9 | 1.771654 | 5.061744 | 0.037507 |
| GO:0007528~neuromuscular junction development | 8 | 1.574803 | 8.248768 | 0.007518 |
| GO:0009888~tissue development | 7 | 1.377953 | 7.578556 | 0.031626 |
| GO:0001502~cartilage condensation | 6 | 1.181102 | 11.81074 | 0.016563 |
| GO:0035987~endodermal cell differentiation | 6 | 1.181102 | 8.959869 | 0.04162 |
| GO:0001894~tissue homeostasis | 6 | 1.181102 | 8.959869 | 0.04162 |
| GO:2001046~positive regulation of integrin-mediated signaling pathway | 5 | 0.984252 | 14.43534 | 0.031858 |
