# Supplementary material for: RA-induced prominence-specific response resulted in distinctive regulation of Wnt and osteogenesis
Source: Life Sci Alliance. 2023 Aug 4;6(10):e202302013. doi: 10.26508/lsa.202302013 (PMC10403638; doi:10.26508/lsa.202302013)
Supplement: Supplementary file 5 [file LSA-2023-02013_TableS5.doc]

**Supplemental Table S5** KEGG pathway enrichment analysis in genes differentially expressed by Prominence.

| **GO Biological Process Term** | **Count** | **GeneRatio** | **Fold.Enrich** | **p.adjust** |
| --- | --- | --- | --- | --- |
| mmu05165: Human papillomavirus infection | 30 | 5.905512 | 3.297316 | 1.64E-06 |
| mmu05200: Pathways in cancer | 30 | 5.905512 | 2.198211 | 0.001912 |
| mmu04510: Focal adhesion | 28 | 5.511811 | 5.542553 | 1.25E-10 |
| mmu04151: PI3K-Akt signaling pathway | 26 | 5.11811 | 2.881554 | 0.000133 |
| mmu04512: ECM-receptor interaction | 19 | 3.740157 | 8.590507 | 3.98E-10 |
| mmu05205: Proteoglycans in cancer | 18 | 3.543307 | 3.493546 | 0.000488 |
| mmu04020: Calcium signaling pathway | 18 | 3.543307 | 2.984071 | 0.00199 |
| mmu04010: MAPK signaling pathway | 17 | 3.346457 | 2.300644 | 0.020479 |
| mmu04974: Protein digestion and absorption | 16 | 3.149606 | 5.894461 | 3.41E-06 |
| mmu04371: Apelin signaling pathway | 14 | 2.755906 | 4.065887 | 0.001072 |
| mmu04921: Oxytocin signaling pathway | 13 | 2.559055 | 3.380647 | 0.006117 |
| mmu04933: AGE-RAGE signaling pathway in diabetic complications | 12 | 2.362205 | 4.727241 | 0.001072 |
| mmu04935: Growth hormone synthesis, secretion and action | 12 | 2.362205 | 4.11596 | 0.002544 |
| mmu04926: Relaxin signaling pathway | 12 | 2.362205 | 3.701173 | 0.005553 |
| mmu05418: Fluid shear stress and atherosclerosis | 12 | 2.362205 | 3.226022 | 0.011744 |
| mmu04310: Wnt signaling pathway | 12 | 2.362205 | 2.792113 | 0.02439 |
| mmu01522: Endocrine resistance | 11 | 2.165354 | 4.706061 | 0.00199 |
| mmu04724: Glutamatergic synapse | 11 | 2.165354 | 3.87313 | 0.006269 |
| mmu04919: Thyroid hormone signaling pathway | 11 | 2.165354 | 3.647198 | 0.009007 |
| mmu04611: Platelet activation | 11 | 2.165354 | 3.529546 | 0.01103 |
| mmu04936: Alcoholic liver disease | 11 | 2.165354 | 3.103998 | 0.020479 |
| mmu05224: Breast cancer | 11 | 2.165354 | 2.977304 | 0.02439 |
| mmu04658: Th1 and Th2 cell differentiation | 10 | 1.968504 | 4.521319 | 0.005357 |
| mmu04657: IL-17 signaling pathway | 10 | 1.968504 | 4.278238 | 0.006269 |
| mmu04713: Circadian entrainment | 10 | 1.968504 | 4.05996 | 0.008587 |
| mmu05146: Amoebiasis | 10 | 1.968504 | 3.718468 | 0.013188 |
| mmu04931: Insulin resistance | 10 | 1.968504 | 3.617056 | 0.014677 |
| mmu05017: Spinocerebellar ataxia | 10 | 1.968504 | 2.821816 | 0.049375 |
| mmu04912: GnRH signaling pathway | 9 | 1.771654 | 3.978761 | 0.014939 |
| mmu05222: Small cell lung cancer | 9 | 1.771654 | 3.850414 | 0.017048 |
| mmu05145: Toxoplasmosis | 9 | 1.771654 | 3.25535 | 0.037963 |
| mmu05031: Amphetamine addiction | 8 | 1.574803 | 4.613056 | 0.014545 |
| mmu04920: Adipocytokine signaling pathway | 8 | 1.574803 | 4.483111 | 0.015206 |
| mmu05412: Arrhythmogenic right ventricular cardiomyopathy | 8 | 1.574803 | 4.133778 | 0.021006 |
| mmu04970: Salivary secretion | 8 | 1.574803 | 3.744716 | 0.03314 |
| mmu04211: Longevity regulating pathway | 8 | 1.574803 | 3.536676 | 0.0426 |
| mmu05410: Hypertrophic cardiomyopathy | 8 | 1.574803 | 3.497812 | 0.04394 |
| mmu05414: Dilated cardiomyopathy | 8 | 1.574803 | 3.38618 | 0.049447 |
