# Supplementary material for: RA-induced prominence-specific response resulted in distinctive regulation of Wnt and osteogenesis
Source: Life Sci Alliance. 2023 Aug 4;6(10):e202302013. doi: 10.26508/lsa.202302013 (PMC10403638; doi:10.26508/lsa.202302013)
Supplement: Supplementary file 10 [file LSA-2023-02013_TableS10.doc]

**Supplementary Table 10 The primers for RT-qPCR.**

| Primer name | Primer sequences (5'-3') |
| --- | --- |
| Fzd5-F | TCTTCCTGTGCTCTATGTACAC |
| Fzd5-R | TAATCCATACACAGAACCTCGG |
| Fzd9-F | CATGAAGACGGGAGGCACCAATAC |
| Fzd9-R | GCCGCCAGAAGTCCATGTTGAG |
| Dvl3-F | TATGGCTTTCCCTTACCAGTAC |
| Dvl3-R | GTGTAGTGTGGTCTGACTCG |
| Foxn3-F | AGGCTCTGAAAAAGACACCTTA |
| Foxn3-R | CTCGCTCCATTTTGTATCACTC |
| Itgb1-F | TGCTCCCACTTCAATCTCACCAAAG |
| Itgb1-R | CCAGCAGTCATCAATGTCCTTCTCC |
| Bmp7-F | GATCCTGTCCATCTTAGGGTTG |
| Bmp7-R | GTTGTACAGGTCCAACATGAAC |
| Alp l-F | GCACCTGCCTTACCAACTCT |
| Alp l-R | GTGGAGACGCCCATACCATC |
| Runx2-F | CGGACGAGGCAAGAGTTTCA |
| Runx2-R | GGATGAGGAATGCGCCCTAA |
| Col1a1-F | AAGGTGCCAATGGTGCTC |
| Col1a1-R | ACCAGTGTCTCCTTTGTTGC |
| Sox5-F | AGGCAGGAAATGCGACAGTAC |
| Sox5-R | CTCGGAGGGCAGGTGAGG |
| Sox6-F | TGCGACAGTTCTTCACTGTGG |
| Sox6-R | CGTCCATCTTCATACCATACG |
| Sox9-F | GCTGTGGAGGCTGCTGAATGAG |
| Sox9-R | CCTGCGTGGTTGGTACTTGTAGTC |
| Col2-F | GTGGAGCAGCAAGAGCAAG |
| Col2-R | CGGAGGAAAGTCATCTGGAC |
| Col10a1-F | TTCTGCTGCTAATGTTCTTGACC |
| Col10a1-R | GGGATGAAGTATTGTGTCTTGGG |
| GAPDH-F | GGCAAATTCAACGGCACAGTCAAG |
| GAPDH-R | TCGCTCCTGGAAGATGGTGATGG |
